# Oxygen Consumption Constrains Food Intake in Fish Fed Diets Varying in Essential Amino Acid Composition

**DOI:** 10.1371/journal.pone.0072757

**Published:** 2013-08-21

**Authors:** Subramanian Saravanan, Inge Geurden, A. Cláudia Figueiredo-Silva, Suluh Nusantoro, Sadasivam Kaushik, Johan Verreth, Johan W. Schrama

**Affiliations:** 1 Aquaculture and Fisheries Group, Wageningen Institute of Animal Sciences (WIAS), Wageningen University, Wageningen, The Netherlands; 2 INRA, UR 1067, Nutrition, Metabolism and Aquaculture (NuMeA), Pôle d′Hydrobiologie INRA, St. Pée-sur-Nivelle, France; Texas Tech University, United States of America

## Abstract

Compromisation of food intake when confronted with diets deficient in essential amino acids is a common response of fish and other animals, but the underlying physiological factors are poorly understood. We hypothesize that oxygen consumption of fish is a possible physiological factor constraining food intake. To verify, we assessed the food intake and oxygen consumption of rainbow trout fed to satiation with diets which differed in essential amino acid (methionine and lysine) compositions: a *balanced vs*. an *imbalanced amino acid diet*. Both diets were tested at two water oxygen levels: *hypoxia vs. normoxia*. Trout consumed 29% less food under hypoxia compared to normoxia (*p*<0.001). Under both hypoxia and normoxia trout significantly reduced food intake by 11% and 16% respectively when fed the imbalanced compared to the balanced amino acid diet. Oxygen consumption of the trout per unit body mass remained identical for both diet groups not only under hypoxia but also under normoxia (*p*>0.05). This difference in food intake between diets under normoxia together with the identical oxygen consumption supports the hypothesis that food intake in fish can be constrained by a set-point value of oxygen consumption, as seen here on a six-week time scale.

## Introduction

The majority of animals, including fish, show a reduction in food intake when the food has an imbalanced essential amino acid composition [1], [2], [3]. It is not clear which physiological factor (constraint) forces an animal to compromise its food intake when confronted with a dietary amino acid deficiency [4]. In rodents, physiological factors such as changes in postprandial blood and brain concentration of free amino acids and ammonia (liberated by deamination) down regulate food intake when fed with amino acid deficient diets [5], [6], [7], [8], [9]. Intake of an amino acid imbalanced diet results in a less efficient use of amino acids for protein synthesis. The most limiting amino acid is best utilised while others get wasted, which leads to a greater ammoniagenesis and ureagenesis. The carbon remnants of these amino acids are either oxidized or used in *de novo lipogenesis*, which increases the oxygen consumption [10], [11]. Compared to terrestrial animals, oxygen is a relatively scarce resource for fish and moreover, gill breathing in water requires more energy than air-breathing [12].

**Figure 1:**
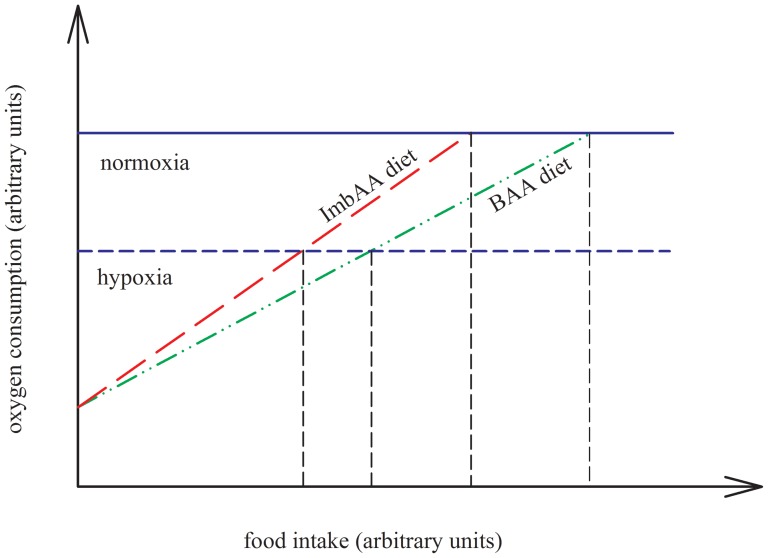
Conceptual illustration of the hypothesis tested in the present study. It is hypothesized that under non-limiting water oxygen level (normoxia) food intake of fish fed a diet deficient in essential amino acids is compromised by a physiological constraint in oxygen consumption. To test the hypothesis, fish were fed under normoxia with diets contrasting in essential amino acids (lysine and methionine) composition: an imbalanced (ImbAA) *vs.* a balanced amino acid (BAA) diet. The difference in amino acid composition of the diet is expected to create differences in metabolism, which will alter the amount of oxygen consumption per unit of food intake. This amount of oxygen consumption per unit of food is higher at the ImbAA diet than at the BAA diet (as indicated by the differences in the slope of lines). As such: 1) If oxygen consumption is constraining the food intake, then the food intake between ImbAA and BAA fed fish will be different but the oxygen consumption will be similar; 2) If oxygen consumption is not constraining the food intake, then food intake between ImbAA and BAA fed fish will be different but also the oxygen consumption. Further, to verify the effect of dietary amino acid induced changes in oxygen consumption on food intake, we measured food intake under limiting water oxygen level (hypoxia) as a positive control.

Therefore, we hypothesize that oxygen consumption is one of the possible physiological factors (constraints) which can limit food intake in fish. We propose that even at normoxic conditions and in the absence of other potential constraints on food intake, food intake in fish can be constrained by a set-point value of oxygen consumption (on a time scale of larger than weeks). In the present study, we assessed the food intake and oxygen consumption of rainbow trout fed diets, which were contrasting in their composition of two essential amino acids (methionine and lysine; *a balanced vs. an imbalanced diet*). Both diets were tested at two different water dissolved oxygen (DO) concentrations (*hypoxia vs. normoxia*). The conceptual illustration of the hypothesis tested is shown in figure 1. The dietary deficiency of both lysine and methionine in the current study was used with the purpose to create a contrast in oxygen consumption per unit food intake (i.e. a difference in slope as depicted in figure 1). In addition to normoxia, we measured food intake and oxygen consumption under hypoxia as a positive control in order to verify the effect of dietary amino acid induced changes in oxygen consumption on food intake, at limiting water DO levels.

## Results

### Food Intake and Growth

**Figure 2:**
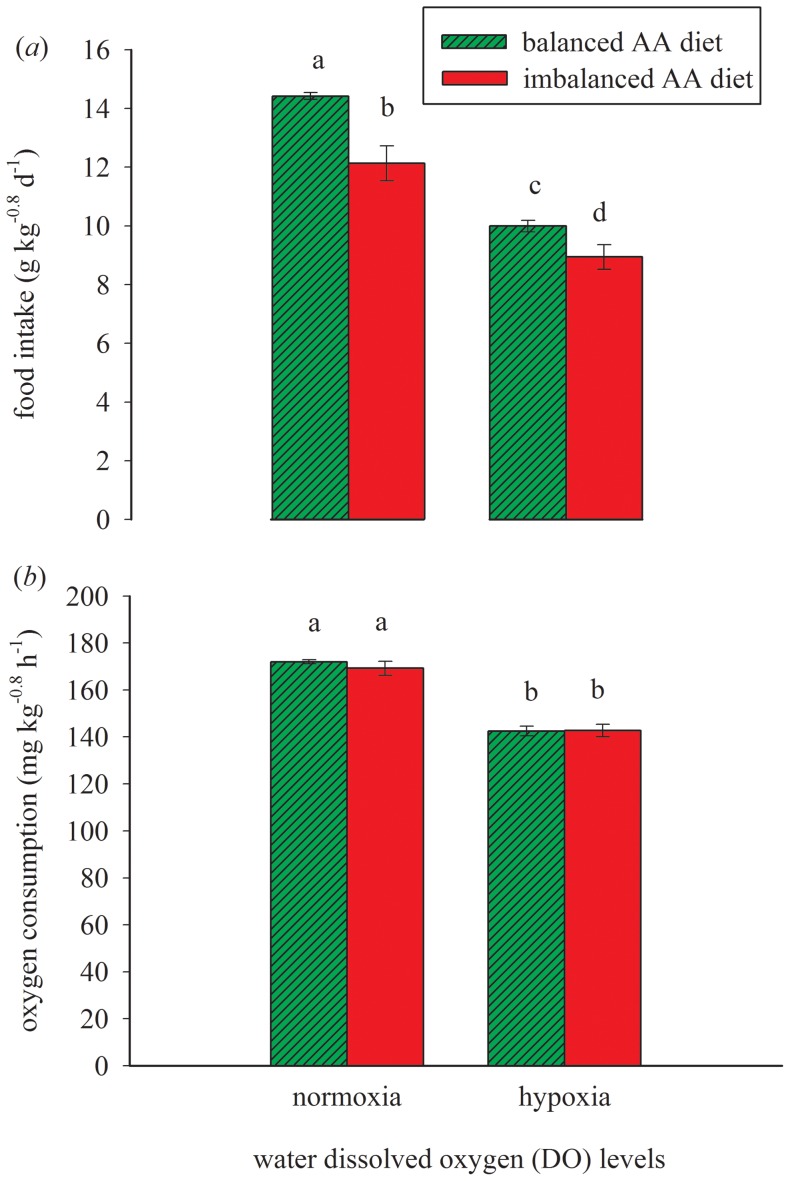
Effect of diet and dissolved oxygen on food intake and oxygen consumption in rainbow trout. Rainbow trout fed to satiation with a balanced amino acid diet and an imbalanced amino acid diet at two levels of water dissolved oxygen (DO): hypoxia *vs.* normoxia. (a) Food intake was affected by dietary amino acid composition (*p*<0.001), water DO level (*p*<0.001), and the interaction between both factors (*p* = 0.02). (b) Oxygen consumption was affected by water DO level (*p*<0.001) but unaffected by dietary amino acid composition (*p* = 0.36) and the interaction between both factors (*p* = 0.31). Values are mean±SD (*n* = 3, group of 30 fish tank^-1^).

Food intake of trout was clearly affected by the diet (*p*<0.001; figure 2*a*). Under hypoxia, the food intake of trout was 11% lower when fed the imbalanced compared to the balanced amino acid diet. Similarly, under normoxia, the food intake was 16% lower for fish fed the imbalanced compared to the balanced diet. Regardless of the dietary amino acid composition, trout kept under hypoxia showed a 29% lower food intake than under normoxia (figure 2*a*). The difference in food intake of trout between both diets was greater under normoxia than hypoxia, as indicated by the significant interaction between water DO level and diet. The digestible protein intake and digestible energy intake of trout paralleled the food intake. The intakes of specific amino acids (glutamic acid, lysine, and methionine) of trout were in line with the created contrast in amino acids between the diets (table 1).

**Table 1:** Fish performance, metabolic parameters and body composition of rainbow trout fed to satiation with balanced amino acid diet and imbalanced amino acid diet at two levels of water dissolved oxygen: hypoxia *vs.* normoxia for 42 days.

	Normoxia			Hypoxia			*P* value		
	Balanced diet	Imbalanced diet		Balanced diet	Imbalanced diet	Pooled SEM	Water DO level	Diet	Water DO x Diet
***Fish performance***									
Initial body weight (g)	53.0	52.0		53.3	51.9	0.72	0.844	0.143	0.828
Final body weight (g)	144.4^a^	109.5^b^		110.8^c^	92.5^b^	2.52	<0.001	<0.001	0.011
Growth (g kg^−0.8^ d^−1^)	15.3^a^	10.8^b^		10.7^b^	8.2^c^	0.29	<0.001	<0.001	0.010
Food intake (g DM fish^−1^ d^−1^)	2.1^a^	1.5^b^		1.3^c^	1.1^d^	0.04	<0.001	<0.001	0.006
Digestible energy intake (kJ kg^−0.8^ d^−1^)	259^a^	213^b^		185^c^	162^d^	4.1	<0.001	<0.001	0.021
Digestible protein intake (g kg^−0.8^ d^−1^)	5.4^a^	4.4^b^		3.7^c^	3.2^d^	0.08	<0.001	<0.001	0.014
Glutamic acid intake (mg kg^−0.8^ d^−1^)	1400^a^	1399^a^		970^b^	1031^b^	24.9	<0.001	0.263	0.249
Lysine intake (mg kg^−0.8^ d^−1^)	287^a^	125^b^		199^c^	92^d^	2.5	<0.001	<0.001	<0.001
Methionine intake (mg kg^−0.8^ d^−1^)	131^a^	74^b^		91^c^	55^d^	1.4	<0.001	<0.001	<0.001
***Metabolic parameters***									
Total ammonia nitrogen loss (mg N per g DPI)	50	73		49	71	6.2	0.779	0.006	0.965
Protein retention efficiency (%)*	44.9	34.3		45.2	33.9	0.48	0.998	<0.001	0.482
***Final body composition*** **									
Protein (g kg^−1^)	164.3^a^	145.1^b^		164.2^a^	140.1^b^	0.94	0.028	<0.001	0.033
Fat (g kg^−1^)	108.0	123.0		99.2	113.1	3.36	0.024	0.003	0.882

SEM, Standard error mean; DO, dissolved oxygen; DM, dry matter; DPI, digestible protein intake.

Mean values in a row with unlike superscript were significantly different and assigned only if the interaction effect was significant (*p*<0.05).

* Protein retention efficiency (%)  =  (Wet protein gain/protein intake in dry weight) ×100.

** Initial body composition (g kg wet weight^−1^): 157, protein and 80.5, fat.

At end of the 6 weeks, the survival of fish was above 98% and was not different between treatments (*p* = 0.73). Trout fed the amino acid balanced diet had better growth than trout fed the amino acid imbalanced diet. Likewise, trout kept under normoxia had a larger final body weight and higher growth compared to fish kept under hypoxia (table 1).

### Body Composition and Nutrient Utilisation

As expected, the difference in amino acid composition of the diet altered the nitrogen utilisation by the rainbow trout as well as their body composition (table 1). The total ammonia nitrogen excretion per unit digestible protein intake was affected by the diet (*p* = 0.006), being 45% higher in trout fed the imbalanced than in those fed the balanced amino acid diet. The protein retention efficiency was thus significantly lower (24%) in trout fed the imbalanced than in those fed the balanced amino acid diet. Both nitrogen excretion and retention efficiency were unaffected by the water DO level and its interaction with diet (*p*>0.05; table 1). Both under hypoxia and normoxia, trout fed the amino acid imbalanced diet showed 14% more body fat than fish fed the balanced diet, despite their lower food intake.

### Oxygen Consumption

The oxygen consumption of trout was lower under hypoxia than normoxia, which was due to the applied contrast in water DO levels (figure 2*b*). Under hypoxia, the oxygen consumption of trout was identical at the balanced (142.5 mg kg^−0.8^ h^−1^) and imbalance amino acid (142.6 mg kg^−0.8^ h^−1^) diet (*p* = 0.36). Despite the differences in food intake under normoxic conditions, the oxygen consumption of trout fed the balanced (171.9 mg kg^−0.8^ h^−1^) or the imbalanced amino acid (169.2 mg kg^−0.8^ h^−1^) diet were equal (*p* = 0.36). There was no significant interaction effect between water DO level and diet on the oxygen consumption of trout (*p* = 0.31).

## Discussion

We found that irrespective of the water DO levels, the rainbow trout fed the amino acid imbalanced diet did not increase food intake to compensate for the inadequate amount of lysine and methionine supplied by this diet. Growth was reduced in trout fed the amino acid imbalanced diet, in agreement with data from most of the amino acid requirement studies in fish [13], [14], [15], [16]. In mammals, the capacity to sense specific nutrients in the diet has been well documented [9]. Similarly, the changes in diet selection pattern when fed diets differing in amino acid composition have been reported in some fish species [3], [17]. The difference in food intake between the amino acid balanced and imbalanced diet groups validates that trout are able to detect the presence of specific dietary essential amino acid. Nevertheless, our aim was to understand the mechanisms involved in the reduced food intake of trout fed the imbalance compared to the balanced amino acid diet, and this under conditions of contrasting water DO levels (hypoxia *vs.* normoxia).

Regulation of food intake in fish is influenced by various physiological conditions, which interact with dietary and environmental factors [18]. Among environmental factors, the water DO level is known to influence food intake and growth of fish [19], [20], [21], [22], [23], [24], [25], [26], as confirmed in the current study by the lower food intake in trout under hypoxia compared to normoxia. Reduced food intake under limited DO conditions (hypoxia) has been explained by a reduced metabolic scope of oxygen for aerobic activities and metabolism, including those related to food processing [25], [27]. Our data further show an effect of dietary amino acid composition on food intake under hypoxia. The lower food intake in trout fed the imbalanced amino acid diet relative to the balanced diet, while accompanied by similar oxygen consumption, is possibly caused by the imposed limitation in oxygen availability combined with increased oxidative metabolism for nutrient processing when fed the amino acid imbalanced diet. Indeed, averaged over both hypoxia and normoxia, the oxygen consumption per gram digestible protein intake (DPI) of trout fed the amino acid imbalanced diet was 992 mg O_2_ per g DPI, which was about 18% higher than when fed the amino acid balanced diet (841 mg O_2_ per g DPI). The higher oxygen consumption in the amino acid imbalanced diet groups is likely to be attributed to an obligatory increase in amino acid deamination and the further processing of amino acid carbon skeleton towards *de novo lipogenesis* [11]. This is confirmed in the current study by the higher ammonia excretion, lower nitrogen retention and increased body fat content of trout fed the imbalanced compared to the balanced amino acid diet. Thus, the higher oxygen demand for metabolism along with the imposed limitation of oxygen availability under hypoxia is likely to constrain the food intake of trout, explaining the lower intake of imbalanced amino acid diet.

**Figure 3:**
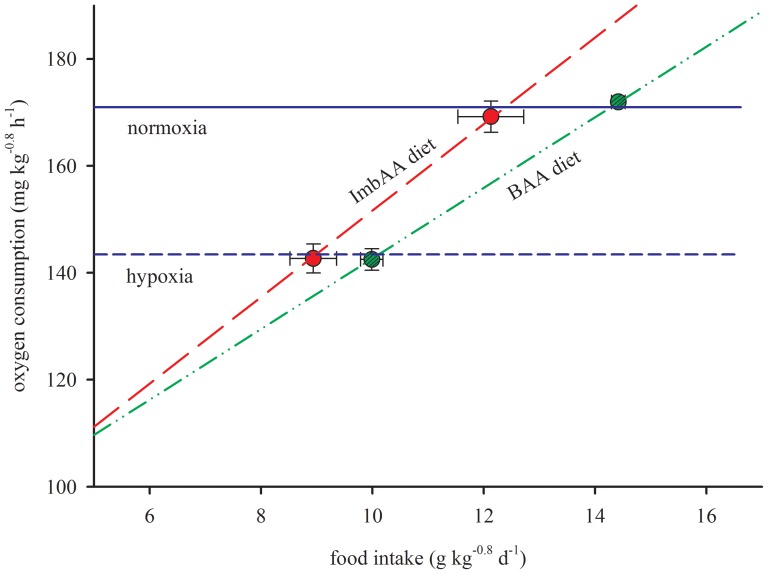
Food intake and oxygen consumption of trout in relation to the proposed hypothesis (figure 1). The measured food intake and oxygen consumption of rainbow trout fed to satiation with a balanced amino acid diet and an imbalanced amino acid diet at two levels of water dissolved oxygen: hypoxia *vs.* normoxia. Values are mean±SD (*n* = 3).

Of interest, exactly similar observations were made under normoxia as under hypoxia. Even at non-limiting water DO level, rainbow trout reduced their food intake when fed the imbalanced amino acid diet compared to the balanced diet. Moreover, even under normoxia, trout of both dietary groups displayed identical oxygen consumption, although the oxygen consumption was higher than those seen under hypoxia. Unlike under hypoxia, this diet-specific reduction in food intake points toward a constraint imposed by factors other than by the availability of water DO (i.e., supply of oxygen to the fish). As mentioned above, the metabolic handling of the amino acid imbalanced diet under normoxia also requires higher oxygen consumption per unit of digested protein intake compared to the balanced diet. As such, the equal oxygen consumption of trout in both the dietary groups together with the differences in voluntary food intake (figure 3) suggests that the oxygen consumption may act as a physiological factor constraining food intake. This is in conformity with our hypothesis as shown in figure 1, that there is set-point oxygen consumption over a period of weeks. The question remaining is what dictates the set-point oxygen consumption. An increased oxidative metabolism has been suggested to exert a negative effect on the organism with cellular damage, for instances due to the production of reactive oxygen species [28]. This implies the oxygen consumption as an intrinsic cost of food intake [29], [30]. Therefore, reducing food intake of a high oxygen demanding diet, such as the amino acid imbalanced diet might be a strategy of fish to avoid the negative effects related to increased oxygen use. In support of the present results, a previous study [31] in Nile tilapia similarly showed an equal oxygen consumption (in three out of four dietary treatments) together with altered intake levels of diets which differed in the amount of oxygen consumed per unit of digestible energy intake, created by changes in dietary macronutrients. This and the present findings (figure 3) of diet-induced differences in food intake in concert with similar oxygen consumptions, obtained at normoxia in both species, suggest that physiological constraints related to oxygen consumption might play a role in the control of food intake in fish, even under non-limiting DO conditions.

## Materials and Methods

### Ethics Statement

This study complied with the Dutch law on experimental animals and was approved by the ethical committee for animal experiments of Wageningen University (DEC: 2011016.d).

### Fish and Housing Conditions

Three hundred and sixty juvenile rainbow trout (*Oncorhynchus mykiss*; Mohnen Aquaculture, Germany) with an average initial body weight of 52.5 g±1.2 g (mean±SD) were randomly allocated among 12 tanks (30 fish tank^−1^) of the aquatic metabolism unit (De Haar Vissen, Wageningen University). The tanks were connected to a closed recirculation system, equipped to adjust and monitor water flow and oxygen concentration in the inflow water of each tank of the fish housing facility, as for details reported elsewhere [31]. Throughout the experiment, fish were reared under the following conditions: photoperiod (12 L∶12 D), water temperature (13.8±0.4°C), pH (7.5±0.3), conductivity (2.9±0.2 mS cm^−1^), nitrite (<0.08 mg N l^−1^), nitrate (<32 mg N l^−1^), total-NH_3_-N (<0.15 mg N l^−1^).

### Treatments

Treatments were designed in a 2 by 2 factorial setup with dietary amino acid composition and water dissolved oxygen (DO) levels as main factors, each consisting of two levels; an amino acid ‘balanced’ *vs.* ‘imbalanced’ diet and ‘normoxia’ *vs.* ‘hypoxia’, respectively. The contrast in dietary amino acid composition was made sufficiently large in order to alter the oxygen consumption required per unit of food, being ‘high’ with an imbalanced (deficient) amino acid diet and ‘low’ with a balanced (adequate) amino acid diet. Thus, two diets differing in the limiting amino acid content (i.e. lysine and methionine) were prepared: a balanced amino acid diet which meet the lysine (5.1 g per 16 g N) and methionine (2.4 g per 16 g N) requirement for rainbow trout [32], and an imbalanced amino acid diet which was 47% deficient in lysine (2.7 g per 16 g N) and 31% deficient in methionine (1.6 g per 16 g N) requirement. The ingredient composition of the experimental diets is given in table 2. The diets were prepared (Research Diet Service B.V., Wijk bij Duurstede) by extrusion process (Clextral BC45, twin screw extruder) with a 3 mm die, dried (70°C for 3 h), vacuum coated with oil and stored at 4°C. The dietary nutrient compositions are shown in table 3.

**Table 2:** Ingredient composition of the experimental diets.

Ingredients (%)	Balanced diet	Imbalanced diet
Wheat gluten	26	26
Soy protein concentrate	14	14
Lysine HCl	1.3	–
DL-methionine	0.4	–
L-glutamic acid	–	1.7
Gelatinized maize starch*	11	11
Wheat	28.5	28.5
Fish oil†	11.8	11.8
Mono-calcium phosphate	3	3
Calcium phosphate	1	1
Diamol‡	2	2
Vitamin-mineral premix§	1	1

* Gelatinised maize starch (Merigel® 100; Amylum Group).

† Fish oil (999 Fish Oil; Triple Nine Fish protein).

‡ Diamol (acid-insoluble ash, as inert marker for digestibility measurement) – Diamol GM; Franz Bertram.

§ Vitamin-mineral premix composition is reported elsewhere [31].

**Table 3:** Analysed nutrient and amino acid composition of the amino acid imbalanced and balanced diets.

	Balanced diet	Imbalanced diet
Dry matter (DM; g kg^−1^)	976	965
Crude protein (g kgDM^−1^)	388	377
Crude fat (g kgDM^−1^)	144	137
Starch (g kgDM^−1^)	296	301
Ash (g kgDM^−1^)	73	72
Digestible energy* (kJ gDM^−1^)	17.95	17.52
**Amino acid composition** (% crude protein)		
Arginine	3.94	4.11
Histidine	1.80	1.88
Isoleucine	3.32	3.53
Leucine	6.03	6.37
***Lysine***	***5.13*** †	***2.73***
***Methionine***	***2.35*** †	***1.62***
Phenylalanine	4.23	4.46
Threonine	2.53	2.68
Tryptophan	0.77	0.85
Valine	3.58	3.82
Cysteine	1.52	1.64
Tyrosine	2.58	2.73
Glutamic acid	25.03	30.58

* Digestible energy determined under normoxia treatment of the present study [31]

† For rainbow trout the estimated requirement for lysine, vary from 4.5 to 6.3% crude protein [32] and for methionine, it varies from 1.8 to 2.14% crude protein depending on level of cysteine [13], [32].

The difference in dissolved oxygen (DO) level in the water was created by adjusting the rate of water flow into the tanks. The water volume was kept constant at 200 l in all tanks. For the normoxia groups, the rate of water inflow into tank (mean±SD) was kept at 7.9±0.03 l min^−1^ with mean water DO level of 10.2±0.2 mg l^−1^, and the mean DO content in the outflowing water remained above 8.0 mg l^−1^. When necessary, pure oxygen was injected into the inflow water in order to maintain outflow DO content above 8.0 mg l^−1^. The hypoxia condition was created by reducing the rate of water inflow (1.94±0.02 l min^−1^) with a mean DO content of 9.8±0.2 mg l^−1^, and the mean DO content in the outflowing water remained below 6.0 mg l^−1^ and above 4 mg l^−1^. The level of DO content for the hypoxia treatment was decided based on the reported incipient DO level of about 6.0 mg l^−1^ for rainbow trout [33]. The applied DO level in hypoxia treatment is expected to reduce food intake but with very minimal level of discomfort for rainbow trout. The welfare of fish was assessed daily by observing the food intake (at tank level) and general behaviour of fish.

### Experimental Procedure

The treatments were randomly assigned among 12 tanks to have triplicates for each treatment group. During the experimental period of 6 weeks, fish were hand-fed with their respective diets twice daily to apparent satiation for an hour (09.00–10.00 and 16.00–17.00 hrs). At each feeding session, feed given and uneaten feed were recorded; in addition, uneaten pellets were collected and counted to determine the food intake accurately. Faeces were collected to determine nutrient digestibility in a similar way as described earlier [31]. The oxygen consumption of fish was monitored for the entire experimental period. The concentration of oxygen in the inlet and outlet of each tank was automatically measured at 5 min intervals using an electrode (WTW-Trioximatic® 700 IQ, WTW GmbH, Weilheim, Germany) and data were recorded in a personal computer using interface (HTBasic, Version 9.5, TransEra Corp.). The oxygen measurement was performed in a continuous cycle of 2 consecutive days (48 h; from 08.00 to 08.00 hrs) in a set of four tanks comprising all treatments. Consequently, in 6 days, oxygen concentrations were measured in all the 12 tanks. The oxygen electrode was calibrated once every week. In addition, one continuous 48 h (from 08.00 to 08.00 hrs) measurement of total ammonia nitrogen was performed in all the 12 tanks. The water was continuously sampled at 3 min intervals from a common inlet and outlet of each tank using an auto-sampler (SANplusSYSTEM, Skalar, The Netherlands) and the concentration of total ammonia nitrogen was determined with colorimetric method [34] following the manufacturer's (Skalar) protocol. The oxygen consumption (mg kg^−0.8^ h^−1^) [31] and the total ammonia nitrogen excretion (mg N kg^−0.8^ d^−1^) [35] of fish were calculated using the formula as described previously. Fish were weighed under sedation (0.25 ml l^−1^, 2-phenoxy ethanol) at the start and end of the experiment 36 h after the last meal. Fish used to determine initial (15 fish) and final body composition (8 fish tank^−1^), and the remaining fish at the end of experiment were euthanized by an overdose of anaesthesia (1 ml l^−1^, 2-phenoxy ethanol). Fish samples for body composition were stored at −20°C until further analysis.

### Chemical Analysis

Whole fish samples were pooled per tank, ground, and subsequently freeze-dried before analysis. Analyses of fish were done in triplicates for dry matter (105°C for 24 h), crude protein (Kjeldahl; N x 6.25), crude fat (Soxhlet; 40–60°C) and energy (Bomb calorimetry) as described previously [31]. Nutrient compositions of the diets were determined using the same methods. The amino acid composition of the diets were analysed (AGROBIO, Rennes, France) in an amino acid analyser (Biochrom 30; Pharmacia Biochrom Ltd) according to standard methods [36].

### Calculation and Statistical Analysis

All parameters of fish growth performance, food intake and body composition were calculated as per formulae mentioned earlier [31]. Values were expressed as mean±SD. Two-way ANOVA was used to assess the effect of dietary amino acid composition (diet), water DO level and their interaction (PROC GLM; SAS 9.2, SAS Institute) and was followed by post-hoc Tukey test, if interaction was significant (*p*<0.05). Normal distributions of the residuals were verified using Kolmogorov-Smirnov test (PROC UNIVARIATE).
